# Histone variant macroH2A1 deletion in mice causes female-specific steatosis

**DOI:** 10.1186/1756-8935-3-8

**Published:** 2010-04-01

**Authors:** Mathieu Boulard, Sébastien Storck, Rong Cong, Rodrigo Pinto, Hélène Delage, Philippe Bouvet

**Affiliations:** 1 Université de Lyon, Ecole Normale Supérieure de Lyon, Laboratoire Joliot-Curie (CNRS USR 3010), 46 allée d'Italie, 69364 Lyon cedex 07, France; 2 Université de Lyon, Ecole Normale Supérieure de Lyon, Laboratoire de Biologie Moléculaire de la Cellule, 46 allée d'Italie, 69364 Lyon cedex 07, France; 3 The Institute of Biomedical Sciences and School of Life Sciences, East China Normal University, Shanghai 200241, China

## Abstract

**Background:**

Vertebrate heterochromatin contains a non-allelic variant of the histone H2A called macroH2A1, which has the characteristic of being three times the size of the canonical H2A. The macroH2A1 C-terminal extension can recruit onto chromatin the poly-ADP-ribose polymerase (PARP)1, which is crucial for DNA repair. This led to the speculation that macroH2A1 could be essential for genome surveillance; however, no experimental evidence supported this hypothesis. Because macroH2A1 has been found to be enriched on the inactive X-chromosome in females, it is thought to play a role in sex chromosome dosage compensation through its ability to regulate gene expression. However, more genetic data are needed to further understand the function of macroH2A1 in mammals.

**Results:**

Deletion of the murine gene *H2afy*, which encodes for macroH2A1, resulted in lipid accumulation in liver. Hepatic steatosis caused by *H2afy *disruption occurred specifically in homozygous mutant females. The metabolic disorder constantly affected half of the number of homozygote females. Given the mixed genetic background of the mutants, an unreported genetic modifier is likely to influence the penetrance of the phenotype. In addition, the X-linked *thyroxine-binding globulin *(*Tbg*) gene was specifically upregulated in steatotic livers. Chromatin immunoprecitation indicated that macroH2A1 is enriched at the *Tbg *promoter in wild-type female animals, indicating that increased *Tbg *expression in *H2afy *null mutants is likely to be a direct consequence of the absence of macroH2A1. Furthermore, male mice, which are not prone to the metabolic disorder, had a reduced level of macroH2A1 incorporated into the *Tbg *promoter.

**Conclusions:**

Because TBG is the main carrier of the thyroid hormone T4, which regulates energy metabolism, we propose that overexpression of TBG is responsible for the fat accumulation observed in *H2afy*-deficient liver. Moreover, our results suggest that the sexual dimorphism of the steatotic phenotype is probably due to the different incorporation of macroH2A1 in males and females. In combination with previous studies, our data demonstrate a role for macroH2A1 in regulating homeostasis in a sex-dependent manner, subject to genetic background.

## Background

The histone variant macroH2A1 has the ability to substitute for H2A within the nucleosome and to establish chromatin domains with specific properties, due to its C-terminal non-histone macrodomain [1]. Incorporation of macroH2A1 results in the addition of a C-terminal domain of 25 kDa to a histone fold motif, and thus constitutes the largest known nucleosome modification [1]. MacroH2A1 dynamic recruitment onto the inactive X-chromosome (X_i_) upon inactivation in female mammals indicates a possible function in sex-chromosome dosage compensation [2-5]. However, quantities of macroH2A are similar in males and females, suggesting that a role in X inactivation is not the sole function of this variant [6].

The presence of macroH2A1 at promoter regions was shown to inhibit gene expression by *in vitro *transcription analysis of reconstituted nucleosomes containing macroH2A1 [7-9]. However, knockdown studies performed in cultured cells demonstrated that macroH2A1 is required for both transcriptional silencing [10] and induction [11,12]. This dual role of macroH2A1 in transcription was investigated further in a recent study, which showed that 12% of the genes enriched in macroH2A1 escape silencing [13]. Furthermore, the same study showed that a subset of genes is downregulated upon macroH2A1 knockdown, indicating that macroH2A1 could act as a positive regulator of transcription at some loci [13].

*In vivo*, the presence of macroH2A1 is essential for the silencing of a class of endogenous (non-ecotropic) murine leukaemia viruses [14]. In addition, macroH2A1 plays a function in NAD^+ ^catabolism through its role in the two main pathways of NAD^+ ^consumption. First, macroH2A1 binds to poly-ADP polymerase (PARP)-1 and inhibits its enzymatic activity [12,15,16]. PARP-1 is a nuclear enzyme that catalyzes the cleavage of NAD^+ ^into ADP-ribose and nicotinamide, and the subsequent transfer of ADP-ribose units to target proteins [17]. PARP-1 is implicated in chromatin structure modulation, transcription regulation and double-strand break (DSB) DNA repair [17]. Secondly, macroH2A1 binds the metabolite *O*-acetyl-ADP-ribose produced by the NAD^+ ^dependent histone deacetylase Sirt1 [18]. Although all these recent findings provide biochemical evidence indicating that macroH2A1 plays a role in NAD^+ ^catabolism, genetic evidence is needed to identify its function in energy metabolism in mammals.

In this study, we generated loss-of-function animals through targeted disruption of *H2afy*, the gene that encodes macroH2A1. Mice that lacked macroH2A1 displayed no obvious visible phenotype and no increased sensitivity to ionizing radiation, which excludes an essential function for macroH2A1 in genome surveillance. However, we found that macroH2A1-deficient females exhibit liver lipid homeostasis defects that lead to hepatic steatosis. Interestingly, lipid accumulation in the liver was specific to females. We provide evidence of reactivation of the X-linked *thyroxine-binding globulin *(*Tbg*) gene specifically in steatotic livers. We also demonstrate that the histone variant macroH2A1 is specifically enriched in nucleosomes that occupy the *Tbg *promoter in female hepatocytes, indicating that *Tbg *overexpression is a direct consequence of *H2afy *disruption.

## Results

### Targeting the mouse *H2afy *locus

**Figure 1:**
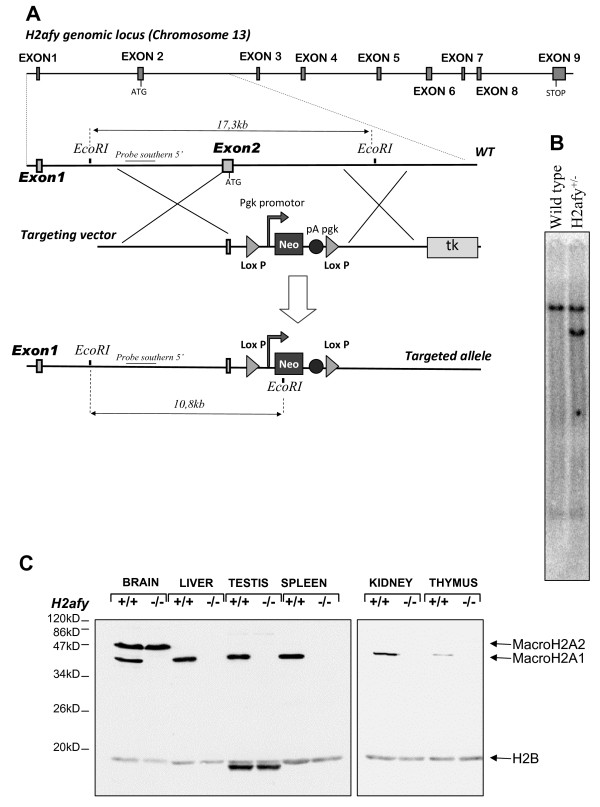
***H2afy *gene-targeting strategy and genotyping of cells and mice**. **(a) **Schematic diagrams of the *H2afy *locus, the targeting vector and the mutant *H2afy *alleles are shown. The exons are numbered. The targeting construct containing a neomycin (Neo) cassette flanked by two loxP sites (triangles) was inserted in place of exon 2, resulting in the deletion of the first coding exon, which encodes amino acids 1 to 57. The targeted locus lacks the translation initiation codon and half of the sequence coding for the histone region, and should therefore function as a null allele. The first 18 nucleotides (non-coding) of exon 2 were not modified by the targeting in order to maintain the correct splicing of the first exon to the second. This mutation affects both splicing isoforms macroH2A1.1 and macroH2A1.2, because they are generated by alternative splicing of exon 6. Probes used for the Southern blot studies and relevant restriction fragments predicted by digestion of the recombinant are shown. The sizes of the diagnostic fragments for the wild-type (WT) and *H2afy *mutant alleles are indicated. **(b) **Southern blot analysis of recombinant embryonic stem cells (digested with *Eco*RI). The blots were hybridized with the probes shown in (a). **(c) **Immunodetection of macroH2A1 and macroH2A2 in *H2afy*^-/- ^and wild-type mice. Nuclear extracts were prepared from various tissues. The protein macroH2A1 was undetectable in the mutant tissue, proving that the targeted mice were null mutants. There was no overexpression of macroH2A2, which was detected only in brain and embryo (data not shown). H2B shows equal protein loading between all lanes. The additional fast migrating bands seen in testis are probably caused by the crossreactivity of the anti-H2B antibody with the testis-specific variant of histone H2B.

We generated an *H2afy *null-mutant mouse strain by homologous recombination in ES cells. Our strategy resulted in the complete disruption of exon 2, which encodes the translation start codon and approximately half of the histone domain (Figure 1a, b).

Mating of heterozygous animals resulted in offspring of all genotypes at the mendelian ratio (95 heterozygous, 68 wild types and 48 homozygous) and the overall sex ratio was close to 1:1. Mice homozygous for the *H2afy *mutation were viable, and survived to adulthood without discernible morphological abnormalities. Both male and female *H2afy*^-/- ^mutants were fertile and produced litter sizes comparable with the wild type.

Absence of the macroH2A1 protein in tissues was confirmed in homozygous mice by immunoblotting using a specific antibody (Figure 1c). To determine whether compensation might be involved, the expression level of *macroH2A2 *(*H2afy2*) was examined in different tissues. MacroH2A2, which is 80% identical to macroH2A1, is encoded by a separate gene located on chromosome 10 [19,20]. Little is known about this second macroH2A subtype, but it was recently shown to be crucial for zebrafish (*Danio rerio*) embryogenesis [21]. In contrast to many human cells and mouse, zebrafish embryos predominantly express *H2afy2*, whereas *H2afy *was not detectable [21].

No overexpression of *H2afy2 *was observed in the absence of macroH2A1 (Figure 1c). Therefore, it is unlikely that the absence of a phenotype in *H2afy-*deficient animals is the result of a functional compensation by *H2afy2*.

### MacroH2A1 is not essential for DNA damage repair

Because macroH2A1 interacts with PARP-1, which is essential for early DNA damage recognition and base excision repair by sensing DNA nicks [17], it has been proposed that macroH2A1 could play a role in the process of DNA repair through its ability to recruit PARP1 onto chromatin [12,16].

**Figure 2:**
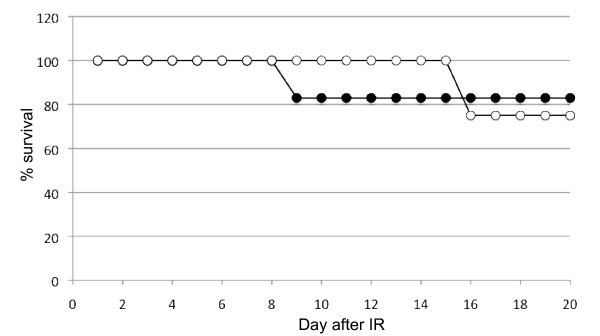
**MacroH2A1 is not required for DNA repair *in vivo***. Survival of 7-week-old *H2afy*^-/- ^mice and littermate controls exposed to 6.5 Gy whole-body gamma irradiation. Four *H2afy*^-/- ^(black circles) and six control mice were used (open circles). *H2afy*-deficient mice were not hypersensitive to DNA damage induced by ionizing irradiation, suggesting that macroH2A1 is not essential for DNA repair.

To determine whether macroH2A1 is essential for DSB repair *in vivo*, we tested the sensitivity of *H2afy*-deficient mice to whole-body ionizing sublethal irradiation. Mice lacking macroH2A1 did not show any increased sensitivity to gamma radiation compared with control littermates (Figure 2). Histological sections through the duodenum did not show any difference in the size of villi between mutant and control mice (data not shown), indicating normal ionizing radiation-induced cell renewal. We conclude that macroH2A1 is not required for accurate DNA break repair *in vivo*.

### Absence of macroH2A1 induces hepatic steatosis in females

**Figure 3:**
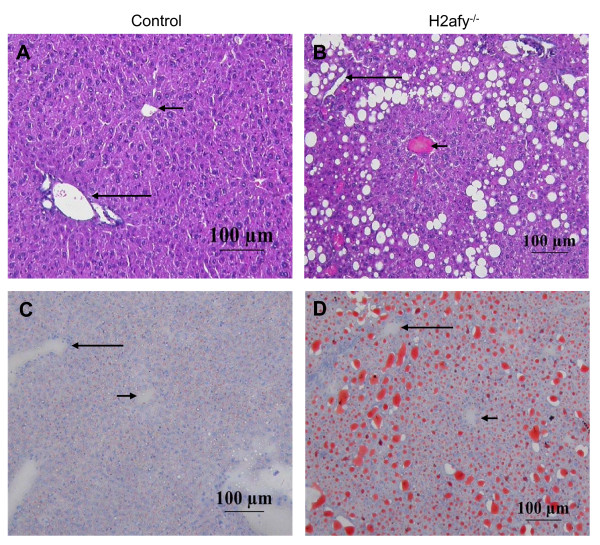
***H2afy *deletion causes female-specific steatosis**. Pathological analysis of the livers from **(b, d) ***H2afy*-targeted and **(a, c) **littermate control mice stained with **(a, b) **hematoxylin and eosin and **(c, d) **oil-red O. **(b) **Microscopic examination of the livers revealed that *H2afy*^-/- ^animals exhibited macrovesicular steatosis mainly in the periportal areas. Strikingly, hepatic steatosis was observed only in *H2afy*^-/- ^females. **(d) **Hepatic steatosis was due to the accumulation of neutral fat in liver, as documented by the positive oil red O staining. The long arrow indicates the portal tract and the short arrow the centrilobular vein.

Histological examination of all major organs, including brain, kidney, spleen, heart, visceral fat and reproductive organs, showed no differences between macroH2A1-deficient mice and control littermates up to 12 weeks of age. However, analysis of liver from *H2afy*^-/- ^females revealed signs of hepatic steatosis (Figure 3, Table 1). Of 17 mutant females, nine displayed periportal hepatocellular macrovacuolation (Figure 3a, b), a phenotype that was not observed in any mutant male or in wild-type male and female mice. We found by staining tissue sections with oil red O that this vacuolation was the result of abnormal triglyceride accumulation in liver (Figure 3c, d). Hence, we conclude that the absence of macroH2A1 induces steatosis.

**Table 1:** Hepatic steatosis incidence

	Control littermates		macroH2A1^-/-^	
	
	Male	Female	Male	Female
Mice presenting hepatic steatosis, n	0	0	0	9

Total examined mice, n	3	7	8	17

Incidence, %	0	0	0	53

### Reactivation of non-ecotropic MLV is not the cause of hepatic steatosis

**Figure 4:**
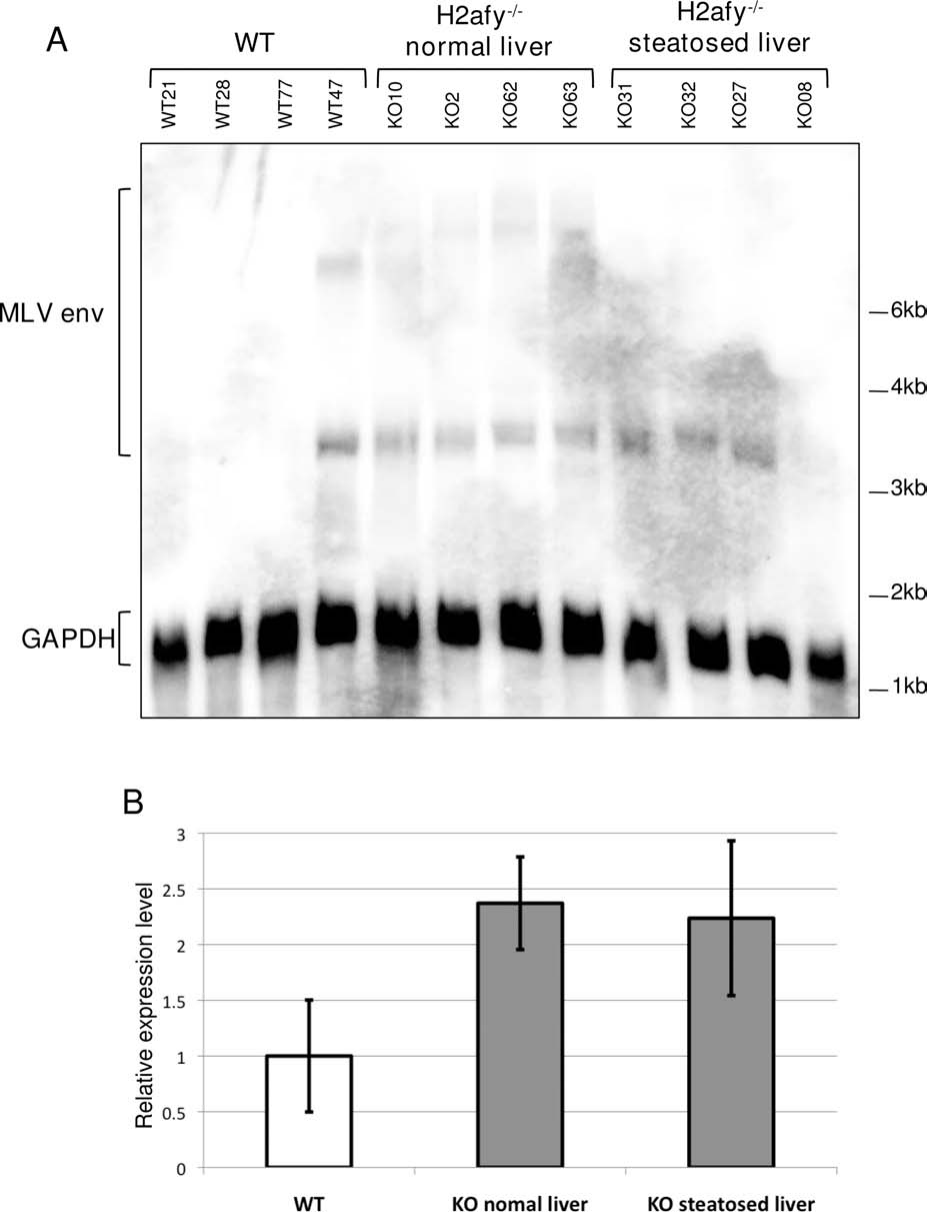
**Steatosis is not linked with endogenous retrovirus reactivation**. **(a) **Northern blot analysis of endogenous MLV*env *expression in liver. Hybridization with a Gapdh probe was used to show equal loading of the lanes. **(b) **Quantification of the intensity of the band from the northern blot. The histogram indicates the relative difference (after normalization for *Gapdh *expression) in the abundance of MLV*env *transcripts with respect to levels observed in wild-type mice, which were set at 1. The error bars represent SEM.

Incorporation of macroH2A1 in chromatin has been shown to be required for the silencing of the endogenous retrovirus murine leukemia virus (MLV) [14]. To explore a possible correlation between MLV overexpression and hepatic steatosis, we assessed MLV *env *transcription in *H2afy*^-/- ^female steatotic livers compared with *H2afy*^-/- ^female healthy livers and wild-type female livers (Figure 4a).

Transcription of MLV elements was not detected above background level in three of four wild-type samples. Surprisingly, an MLV transcript was detected in one wild-type sample (Figure 4a), showing that MLV can also be transcribed in the presence of macroH2A1; this may reflect the segregation of a gene unlinked to *H2afy *that regulates MLV expression. Quantification of the northern-blot data confirmed that MLV silencing is lost in the absence of macroH2A1, as previously reported [14]. MLV expression level, normalized to GAPDH, was upregulated by 2.3-fold in *H2afy*^-/- ^mutants (Figure 4b), but no significant difference was observed between healthy mutant livers and steatotic livers (Figure 4b). We therefore exclude a relationship between hepatic steatosis and MLV overexpression in the absence of macroH2A1.

### Hepatic accumulation of lipids is correlated with upregulation of the X-linked *Tbg *gene

**Figure 5:**
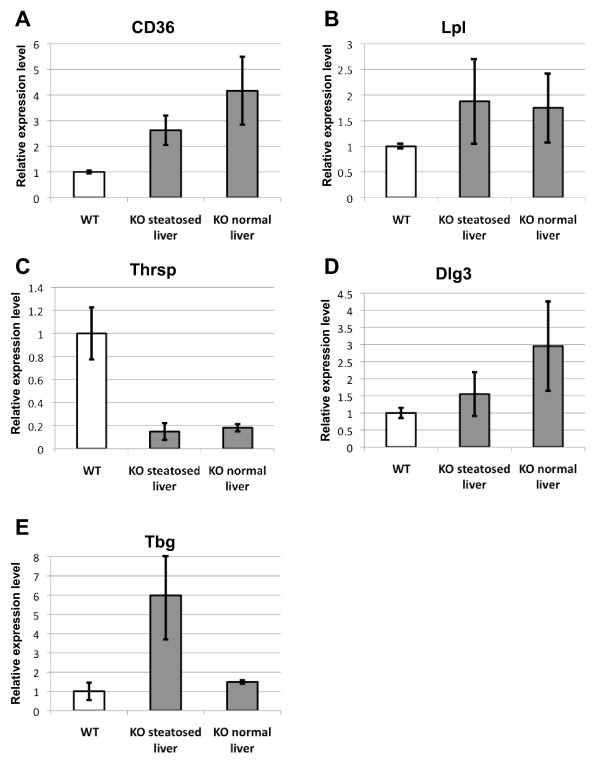
**The steatosis incidence correlates with *Tbg *overexpression**. Real-time quantitative expression analysis of key genes in liver: *CD36, Lpl *and *Thrsp *code for proteins involved in fatty acid metabolism; *Tbg and Dlg3 *are X-linked genes. *Tbg *presented transcriptional upregulation correlating with the incidence of hepatic steatosis. Gene expression was normalized to *Gapdh *levels. Expression levels were corrected against expression measured in control littermate liver, which was defined as 1. The error bars represent SEM.

A major cause of hepatic steatosis is the increased fatty acid flux to the liver caused by a high level of free fatty acids in plasma [22]. To gain further insight into the molecular causes of the triglyceride accumulation in the absence of macroH2A1, we investigated a possible correlation between this phenotype and the expression level of a few genes important for lipid metabolism that have been previously shown to be regulated by macroH2A1 [23] (Figure 5).

The expression level of *lipoprotein lipase *(*Lpl*), which catalyses the hydrolysis of the triacylglycerol component of circulating chylomicrons and very low-density lipoproteins [24], was not affected by the mutation (Figure 5b). Mice lacking macroH2A1 presented an upregulation of *CD36 *(Figure 5a), a fatty acid transporter in muscle and adipose tissue. By contrast, *Thrsp *(thyroid hormone responsive SPOT14 homolog), a regulator of *de novo *lipogenesis, was downregulated in *H2afy*^-/- ^livers compared with controls (Figure 5c) [25]. Thus, our results suggest that *Thrsp *belongs to the recently discovered category of genes that are positively regulated by macroH2A1 [13].

No differences in *Thrsp *or *CD36 *expression were observed between *H2afy*^-/- ^females showing a steatotic liver and those with a healthy liver (Figure 5a, c). Therefore, it is unlikely that the steatotic phenotype observed in *H2afy*^-/- ^females was a consequence of the deregulation of *CD36 *and *Thrsp *expression.

Remarkably, the X-linked *Tbg *gene (also named *Serpina7*) was overexpressed by sixfold in *H2afy*^-/- ^female mice with a steatotic phenotype compared with healthy mutant or wild-type females, which both expressed *Tbg *at the same level (Figure 5e). TBG is a thyroid hormone-binding protein, which carries the majority of the hormone T4 in the serum [26]. Given that thyroid hormones influence general energy metabolism and specifically promote increases in lipid metabolism and counteract lipid accumulation [27], overexpression of *Tbg *in *H2afy*^-/- ^females and its consequences on lipid metabolism could be the origin of the hepatic steatosis we observed.

To test if the overexpression of *Tbg *was a consequence of a global reactivation of X_i _in the absence of macroH2A1, we examined expression of *Dlg3*, another gene located on the X-chromosome (Figure 5d). Although the expression level of *Dlg3 *was slightly elevated in mutant mice, *Dlg3 *did not show any significant upregulation in steatotic liver (Figure 5d). Together, these data indicate that the inactive X-chromosome is not fully reactivated in the absence of macroH2A1, indicating that macroH2A1 is not required for global silencing of X_i_.

### MacroH2A1 occupies the female but not the male *Tbg *promoter

**Figure 6:**
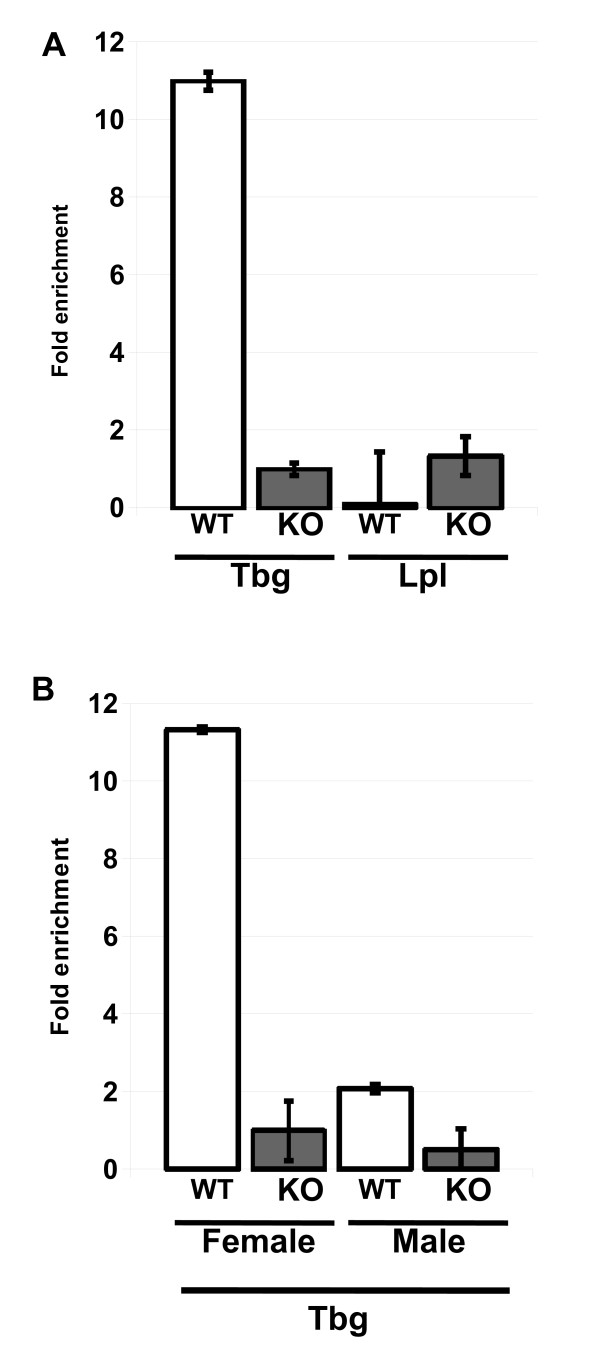
**MacroH2A1 is specifically incorporated into the female *Tbg *promoter**. Chromatin immunoprecipitation (ChIP) analysis of the association of macroH2A1 with key gene promoters involved in lipid metabolism. **(a) **ChIP analysis of macroH2A1 enrichment level on *Tbg *and *Lpl *promoters in females. The graph shows that macroH2A1 was enriched at *Tbg *promoter but not at the *Lpl *promoter in female hepatocytes. **(b) **The *Tbg *promoter in male mice was not significantly occupied by macroH2A1. ChIP using liver chromatin from wild-type (WT) animals and *H2afy*^-/- ^knockout (KO) mice was performed using anti-macroH2A1 antibody. Quantitative PCR was then performed with specific primers for promoter regions of the *Tbg *and *Lpl *genes as indicated in Materials and methods. The results are presented as the fold enrichment in wild-type animals compared with *Tbg *promoter DNA amplified in KO female mice. The graph represents the average of three independent experiments.

To determine if macroH2A1 directly controls the expression of genes most perturbed in *H2afy*^-/- ^liver, we performed chromatin immunoprecipitation (ChIP) experiments to test for the presence of macroH2A1 in nucleosomes that occupied promoters of those genes in livers of wild-type animals (Figure 6).

We first analysed chromatin extracted from female livers (Figure 6a). We did not detect the presence of macroH2A1 above the background level on the *Lpl *gene, which was not deregulated in the absence of macroH2A1 (Figure 5b). By contrast, wild-type female hepatocytes displayed high levels of macroH2A1 incorporated into the sequence upstream of the *Tbg *transcription start site (Figure 6a). This finding strongly suggests that increased expression of *Tbg *measured in *H2afy*^-/- ^livers is a direct consequence of *H2afy *disruption.

To determine the molecular cause of the sexual dimorphism of the steatosis promoted by *H2afy *disruption, we compared macroH2A1 levels on the *Tbg *promoter in male versus female animals (Figure 6b). Consistent with our first analysis (Figure 6a), we found that macroH2A1 is 10-fold enriched on the *Tbg *promoter in females (Figure 6b). Furthermore, our ChIP analysis shows that in contrast to females, the *Tbg *promoter in males has low amounts of bound macroH2A1 (Figure 6b). It should be mentioned that in a previous study [23], this sex-specific difference was not noted. This might be the result of differences in the purification method of macroH2A containing nucleosomes (immunoprecipitation in this study; biochemical purification in the previous study [23]). However, in the previous study, *Tbg *expression was shown to be four times higher in the livers of wild-type adult males than in wild-type adult females [23]. Hence, the sexual dimorphic expression of *Tbg *in wild-type mice is probably caused by the low incorporation of macroH2A1 on the *Tbg *promoter in male hepatocytes.

We conclude that the sexual dimorphism of the phenotype caused by *H2afy *deletion is caused by the sex-dependent incorporation of macroH2A1 at specific loci, as evidenced by the sex-specific incorporation of macroH2A1 on the *Tbg *promoter.

## Discussion

Our study provides novel insights into macroH2A1 functions in X-chromosome inactivation and lipid metabolism. Loss of function of *H2afy *causes hepatic accumulation of lipid specifically in female mice.

Interestingly, a recent report has demonstrated that mice fed with a methyl-deficient diet that induces hepatic steatosis had an elevated level of macroH2A [28]. Although this seemingly contradicts our results, we propose that the elevated level of macroH2A reported in this study reflects a homeostatic response within the liver that is ultimately saturated after prolonged exposure to high levels of lipids. In the light of this study [28], our findings indicate that macroH2A could be incorporated at high levels in chromatin in order to circumscribe hepatic lipid accumulation.

Using a similar approach, Changolkar and colleagues have independently shown that *H2afy*-deficient mice are viable, and that the mutation does not result in any obvious macroscopic phenotype [23]. Those independent data consistently demonstrated that macroH2A1 does not play a fundamental role during development; however, two recent studies have shown that macroH2A1 is preferentially incorporated at developmental gene promoters [13,21]. This apparent contradiction might be explained by the potential existence of redundant chromatin silencing pathways. One hypothesis suggests functional compensation by the polycomb repressive complex (PRC)2, as indicated by its colocalisation with macroH2A1 [29]. This hypothesis is also sustained by the genome-wide colocalisation between macroH2A1 and the trimethylation of lysine 27 of histone H3, the histone posttranslational modification catalysed by PRC2 [13,30]. However, the possible redundant roles of PRC2 and macroH2A1 remain to be experimentally tested.

In addition, the study of Changolkar *et al*. showed that *H2afy *disruption has a minor influence on gene expression, as only seven genes displayed significant abnormal expression in the absence of macroH2A1 [23]. The surprisingly low number of genes affected by the macroH2A1 deletion could be explained either by possible functional compensation by another redundant chromatin repressive mechanism or by the presence of genetic modifiers, as discussed below. Very interestingly, four genes deregulated in *H2afy*^-/- ^livers encode proteins directly involved in fatty acid metabolism, which further supports the role of macroH2A1 in lipid homeostasis control as highlighted in our study. We have independently confirmed the abnormal upregulation of three of these genes in *H2afy*^-/- ^liver. However, we found a decreased level of *Thrsp *in mutant female liver, whereas the study of Changolkar *et al*. concluded that *Thrsp *expression is not affected by *H2afy *disruption in female hepatocytes [23].

The major discordance between these two parallel studies concerns liver steatosis; there is no mention of this in the study from Changolkar *et al*. [23]. This important difference could be explained by the different genetic backgrounds of mice in these two studies. The study by Changolkar *et al*. was performed using a fixed mutation on the C57Bl6 background whereas the mutants we analysed had a genetic background composed of half 129Ola and half C57Bl6 (intercrosses of the F2 generation). Such interference of the genetic background with the phenotype has been extensively documented for many genes [31]. Likewise, variation of the genetic background has been reported to have significant influences on both the quantities of macroH2A1 in steatotic liver and the incidence of steatosis [28].

Additionally, our data reveal that the penetrance of the steatotic phenotype of female progenies of C57Bl/6 × 129Ola mice was very close to half. This consistent penetrance in half the animals over several intercross generations indicates the existence of a genetic modifier present in one of the two mixed genetic backgrounds that is able to modify the penetrance of the phenotype resulting from *H2afy *deletion. Identification of this genetic modifier should expand our knowledge of the metabolic pathway controlled by macroH2A1 in female.

Our study uncovers part of the molecular mechanism by which macroH2A1 regulates lipid homeostasis. Indeed, we show that macroH2A1 silences *Tbg *expression in female liver, as evidenced by the overexpression of *Tbg *in the absence of macroH2A1. We also provide evidence of specific enrichment of macroH2A1 on the *Tbg *promoter, which indicates that macroH2A1 directly regulates *Tbg *transcription.

The consequences of *Tbg *overexpression in mice are unknown. However, studies in patients with amplification of the *Tbg *gene revealed that excess TBG can lead to hypothyroidism and altered metabolism [32,33]. Thus, we propose that elevated levels of TBG, the main T4 hormone carrier, could be the cause of the alteration in lipid metabolism responsible for hepatic steatosis in the absence of macroH2A1.

The reactivation of the X-linked *Tbg *caused by *H2afy *disruption raises the possibility that the remarkable sexual dimorphism of the phenotype might be due to X_i _silencing failure in the absence of macroH2A1. However, we found that expression of *Dlg3*, another X-linked gene, is not affected by *H2afy *disruption. Therefore, X_i _is not entirely reactivated in the absence of macroH2A1. In good agreement with our conclusion, a global analysis of gene expression in *H2afy*^-/- ^liver did not report evidence of X_i _reactivation in the absence of macroH2A1 [23]. This notion was reinforced by a previous study, which reported that *H2afy *knockdown is not sufficient to reactivate the transcription of a reporter gene stably inserted in X_i _[34].

Given the low level of macroH2A1 incorporated into the male *Tbg *promoter, we propose that the sexual dimorphism of the phenotype could be due to the selective incorporation of macroH2A1 on the *Tbg *promoter in female cells. Our measurements show that the amount of macroH2A1 incorporated into the *Tbg *promoter is about fivefold higher in female mice than in male mice. This 5:1 ratio cannot be explained by the twofold difference of the X-chromosome copy number between the sexes. Instead, the difference in macroH2A1 occupancy at the same locus between males and females is probably caused by an unbalanced incorporation of macroH2A1 between the sexes. Specific enrichment on the female X-linked *Tbg *promoter could possibly be a reflection of the specific incorporation of macroH2A1 on X_i _[2-4,35]. The different levels of macroH2A1 on the *Tbg *promoter locus between the sexes might also be a consequence of a more general differential genomic localisation of histone variants between different cell types (for example, male versus female).

## Conclusions

Although much effort has been devoted to understanding the role of macroH2A1 in gene silencing, its biological function remains elusive. Recent advances that uncovered its association with PARP-1 have led to the speculation that macroH2A1 could be involved in DNA repair. However, we report here that *H2afy*^-/- ^mutants are not hypersensitive to DNA DSB, excluding a fundamental role of macroH2A1 in the control of the DNA damage response.

In addition, we have shown that loss of function of *H2afy *causes hepatic accumulation of lipid specifically in females. We also report a correlation between macroH2A1 occupancy on the *Tbg *promoter and its specific overexpression in steatotic liver, which strongly supports our hypothesis that *Tbg *deregulation is the major cause of lipid metabolism dysfunction in the absence of macroH2A1. Thus, our study provides novel insights into macroH2A1 functions in X-linked gene silencing and lipid metabolism.

The importance of chromatin in the control of homeostasis has recently begun to be uncovered with the discovery of the role of histone deacetylase and histone demethylase in the regulation of energy metabolism [36,37]. Our work highlights that histone variants are also involved in the regulation of lipid metabolism.

## Methods

All experiments on mice were carried out with CREA Rhône-Alpes (France) committee approval. Mice were normally housed in cages with littermates of the same sex. Animals were kept in a 12-hour light/dark cycle and fed standard diet rodent chow (SAFE #A04-10 and SAFE #A03-10 for breeders; Perotech Sciences Inc., Toronto, ON, Canada).

### Generation of macroH2A1 targeted mice

*H2afy *was disrupted by replacing exon 2 with a neomycin resistance cassette. Both recombination fragments were first amplified using DNA polymerase (Phusion; New England BioLabs, Ipswich, MA, USA) and appropriate primers (Table 2) on DNA extracted from E14.1 ES cells, then cloned into *Sal*I and *Xho*I restriction sites, respectively, of the pLN-TK targeting vector (Figure 1). The resulting vector was linearized with *Mlu*I and electroporated into E14.1 ES cells (129/Ola strain) as described previoulsy [38]. After antibiotic selection (G418/Geneticin; Gibco-BRL, Grand Island, NY, USA), homologous recombinants were identified by PCR using the set of primers named 'screen2 F' and 'NeoLOXP2 R' shown in Table 2. Homologous recombination was confirmed by Southern blotting and extensive restriction analysis, using flanking and internal probes (data not shown). Two different ES clones heterozygous for the targeted mutation were injected into C57Bl/6 blastocysts and transferred into pseudopregnant foster mothers. One clone gave high-level chimeras and transmitted the disrupted *H2afy *allele to offspring at high frequency when crossed into C57Bl/6 mice. The mice were maintained by intercrosses of the original 129Ola × C57Bl/6 mice. The animals were genotyped by PCR on genomic DNA isolated from finger biopsy, using the primer pairs 'screen F' located in intron 1-2 and 'screen R' located in the targeted portion of intron 2-3, and a third primer complementary to the neo gene 'NeoLOXP2 R' (Table 2). This resulted in the amplification of a 500 bp fragment from the wild-type *macroH2A1 *allele and a 310 bp fragment from the null allele.

**Table 2:** List of primers

Primer name	5' → 3'	
	
	Forward	Reverse
5'' recombination arm	AGCTCGAGCTAGAATGGTGAACAGGTTGGAAGG	TTGTCGACAAACAGATCAGCGAGCTCACTGC

3' recombination arm	AGCTCGAGCTGGTCTGCACACATCCTTTAAGG	ACAGGAGTCAAGATGGTGGCTGGG

Dlg3*	CTATGGGACCAGCATCCAGT	TGTGCTTGCTGCAGTCTCTT

Tbg*	AAGGCTGTGCTACACATTGG	CGGATAACAGGGTGAAGAGG

Lpl*	GAGCGAGAACATTCCCTTCA	TCTGAGTCTCTCCGGCTTTC

CD36*	GCAAAGTTGCCATAATTGAGTCC	GTCTGTGCCATTAATCATGTCG

Thrsp*	GAGTGCATCTGTGGACTTGG	GAGTAACTGCGACATGACACC

GAPDH*	CAACTACATGGTCTACATGTTC	CGCCAGTAGACTCCACGAC

18S*	CGGCTACCACATCCAAGGAA	GCTGGAATTACCGCGGCT

*Reverse transcriptase PCR.

### Immunoblotting

Nuclear proteins were isolated from tissues in accordance with the protocol described previously [39]. Briefly, 10 μg of nuclear proteins were separated by SDS-PAGE and transferred onto nitrocellulose membranes. For the detection of macroH2A1, a polyclonal antibody (Millipore Inc., Billericay, MA, USA) was used. MacroH2A2 was detected using a rabbit polyclonal antibody (cat. no. ab4173; Abcam Inc., Cambridge, MA, USA) at a dilution of 1:2000. Histone H2B was detected using a rabbit polyclonal antibody (cat. no. 07-371; Millipore) at a dilution of 1:5000 [39].

### Southern blotting

For Southern blotting, 10 μg of DNA were digested with 100 U of *Eco*RI, then separated by agarose gel electrophoresis and transferred onto a nitrocellulose membrane, followed by UV crosslinking. Filters were hybridized with a ^32^P-labelled probe located 5' to the sequences in the targeting vector (Figure 1). Membranes were washed at 65°C, twice with 1× saline sodium citrate (SSC) buffer plus 0.1% SDS for 15 minutes each, and twice with 0.1× SSC plus 1% SDS for 30 minutes each, then exposed to a phosphor screen for 24-72 hours. The gel images were acquired with a scanner (Fujifilm 217 FLA-5100; Fujifilm Holdings Corp., Tokyo, Japan) and the pictures were analyzed with Multigauge V.3.0 software (Fujifilm).

### Survival of mice after whole body gamma irradiation

For induction of DNA damage, 7-8-week-old *H2afy*^-/- ^mice and control littermates were exposed to whole-body sublethal ionizing irradiation. Animals were irradiated with a ^137^Cs source at a rate of 0.68 Gy/min in single doses of 6.5 Gy. *H2afy*-deficient mice and seven control littermates were irradiated, and survival was determined. Four days after irradiation, one mutant and one wild type were killed for histopathological analysis of the small intestine.

### Histological analysis

Formaldehyde-fixed, paraffin wax-embedded sections were prepared from liver samples, and were stained with hematoxylin and eosin using standard methods. Frozen liver sections were stained with oil red O. Sections were photographed at 20× magnification.

### Total RNA preparation, reverse transcription and quantitative PCR

Total RNA was isolated using TRIzol reagent (Invitrogen Corp., Carlsbad, CA, USA) and treated with DNaseI, then 5 μg of RNA were reverse transcribed (RevertAid Reverse Transcriptase; Fermentas, Burlington, ON, Canada). Quantitative reverse transcriptase (RT) PCR reactions were performed using the Light-Cycler system (Roche Applied Science; Indianapolis, IN, USA) to determine mRNA levels of *Tbg*, *Lpl*, *CD36*, *Thrsp *and *Dlg3 *(specific primers are listed in Table 2). All cDNAs were normalized using primers to *Gapdh*. Expression levels were corrected against expression measured in control littermate liver, which was defined as 1. cDNA levels from wild-type (n = 2), steatotic *macroH2A1*^-/- ^(n = 4) and healthy *macroH2A1*^-/- ^(n = 3) were compared, and results expressed as mean ± SEM.

### Northern blotting

For Northern blotting, 10 μg of total RNA were denatured and subjected to electrophoresis in a 1% agarose plus 1.9% formaldehyde gel in 1× MOPS buffer, and transferred to a nitrocellulose membrane. The membrane was hybridized with probes derived from radiolabeled extension of random hexamers annealed to the purified 1011 bp fragments produced by MelARVenv digestion by *Nco*I (described in [40]) or *Gapdh *previously amplified from cDNA using the primers listed in Table 2. Quantification of the intensity of the bands was performed using MultiGauge V.3.0 software (Fujifilm). *Gapdh *mRNA served as an internal control, and expression levels of MLV were set to 1 for the wild-type littermate controls. Results are expressed as mean ± SEM.

### Chromatin immunoprecipitation

Mouse liver tissue (1 g) was homogenized in buffer H (250 mM sucrose, 3 mM CaCl_2 _in 20 mM Tris pH 7.4) in the presence of a cocktail of protease inhibitors cocktail (Roche Applied Science, Basel, Switzerland) and spun in a centrifuge at 280 g, for 10 min at 4°C (A-4-81 rotor; Eppendorf North America, Westbury, NY, USA). The resulting pellet was collected and washed three times in phosphate-buffered saline (PBS) and carefully resuspended in a 2.4 M sucrose cushion (2.4 M sucrose, 1 mM MgCl2, 10 mM Tris pH 6.8 and protease inhibitor cocktail). The suspension was centrifuged at 168,000 g for 3 hours at 4°C (SW41 Ti rotor; Beckmann-Coulter Inc., Brea, CA, USA). The nuclear fraction was carefully collected and washed three times in PBS plus protease inhibitor cocktail, then counted and divided into aliquots. Following this, 1.5 × 10^6 ^nuclei were crosslinked for 10 min in 1% paraformaldehyde at room temperature and sheared by sonication to produce DNA fragments averaging 500 bp in length. The sheared chromatins were immunoprecipitated with anti-macroH2A1 antibody (cat. no. 07-219; Upstate Biotech). Precipitated DNA and protein complexes were uncrosslinked and purified by phenol/choloroform extraction. Purified DNA was used as templates for qPCR quantification using *Tbg *or *Lpl *specific primers listed in Table 2.

## Competing interests

The authors declare that they have no competing interests.

## Authors' contributions

MB, SS, RC, RP and PB conceived of and designed the experiments. MB, SS, RC, RP and HD performed the experiments. MB, SS, RC, RP, HD and PB analysed the data. MB and SS generated the *H2afy*-targeted mouse strain. MB, SS and PB coordinated the work and co-wrote the manuscript. All authors read and approved the final manuscript.
